# Genotype by Energy Expenditure Interaction and Body Composition Traits: The Portuguese Healthy Family Study

**DOI:** 10.1155/2014/845207

**Published:** 2014-03-25

**Authors:** D. M. Santos, P. T. Katzmarzyk, V. P. Diego, T. N. Gomes, F. K. Santos, J. Blangero, J. A. Maia

**Affiliations:** ^1^CIFI2D, Faculty of Sports, University of Porto, Rua Dr. Plácido Costa 91, 4200-450 Porto, Portugal; ^2^Pennington Biomedical Research Center, Louisiana State University System, Baton Rouge, LA 70808, USA; ^3^Texas Biomedical Research Institute, San Antonio, Texas 78227-5301, USA

## Abstract

*Background and Aims*. Energy expenditure has been negatively correlated with fat accumulation. However, this association is highly variable. In the present study we applied a genotype by environment interaction method to examine the presence of Genotype x by Total Daily Energy Expenditure and Genotype x by Daily Energy Expenditure interactions in the expression of different body composition traits. *Methods and Results*. A total of 958 subjects from 294 families of *The Portuguese Healthy Family Study* were included in the analysis. TDEE and DEE were assessed using a physical activity recall. Body fat percentages were measured with a bioelectrical impedance scale. GxTDEE and GxDEE examinations were performed using SOLAR 4.0 software. All BC traits were significantly heritable, with heritabilities ranging from 21% to 34%. The GxTDEE and GxDEE interaction models fitted the data better than the polygenic model for all traits. For all traits, a significant GxTDEE and GxDEE interaction was due to variance heterogeneity among distinct levels of TDEE and DEE. For WC, GxTDEE was also significant due to the genetic correlation function. *Conclusions*. TDEE and DEE are environmental constraints associated with the expression of individuals' BC genotypes, leading to variability in the phenotypic expression of BC traits.

## 1. Introduction

The hypothesis that the development of many complex traits are the result of the interplay between genetic background and environmental influences has long been postulated [1] and has been referred to as genotype-by-environment interaction (GxE) [2]. Under such a hypothesis it is expected that genetic effects are dynamically modulated by environmental exposures.

This concept has been used to study obesity for several decades [3], and there is a wealth of data confirming that environmental factors, whether related to nutritional habits and/or physical activity/exercise patterns, play key roles in the accumulation of body fat [4, 5]. However, within a population sharing the same physical activity (PA) habits (in terms of levels and patterns), interindividual variability in body composition is widely observed [6].

Genetic epidemiology research suggests that genetic factors account for 50% to 90% [7] of the total interindividual variability in body fat accumulation. It remains, however, to be explained how environmental and behavioral factors, such as PA, affect the genetic influence on body composition. Twin-based studies have shown that genetic factors influence weight changes following different exercise patterns [8–10]. For example, results from the Swedish Young Male Twins study [5] indicated that for those twins with genetic susceptibility for obesity, engaging in an active lifestyle, had a preventive effect on accumulating fat. Accordingly, Mustelin et al. [11] found an inverse additive genetic correlation between PA and BMI in both genders with correlations of −0.22 and −0.08 for females and males, respectively. More recently, an association study identified significant interactions between individual genes and self-reported PA, suggesting, for example, that the effect of the* FTO rs9939609* polymorphism on body fat accumulation is exacerbated by low levels of PA [12]. Also, it has been shown that PA decreases the impact of* FTO* gene variants on obesity [13]. In a study with Danish and Finnish twin samples [14], the results follow the same trend with an inverse association between PA and WC and BMI and % body fat as well as evidence that PA decreases both genetic and environmental variances of BMI and waist circumference. Moreover, using a GxE model, McCaffery et al. [15] found that BMI is, on average, lower among those individuals that engage in vigorous activities and that vigorous exercise significantly modified the additive genetic component of BMI, confirming the presence of a GxE interaction. Using an animal model, Noland et al. [16] found that even when exposed to a high fat diet, rats with inherited low oxidative capacity were heavier and hypertriglyceridemic when compared to high oxidative capacity rats. As such, it is highly likely that differences in PA patterns and levels may have different impacts on body composition changes within the same population. Accordingly, to better explain why some people become obese while others do not, it is important to understand how PA interacts with genotype and influences its association with body fat.

In the present study, using a nuclear family design, we bring together information on body composition and energy expenditure aiming (1) to estimate the magnitude of the genetic effects on body composition (BC) traits and (2) to examine the Genotype x Total Daily Energy Expenditure (GxTDEE) and Genotype x Daily Energy Expenditure (GxDEE) interactions that may affect the impact of PA on BC traits. Our main hypothesis is that the genetic regulation of BC is affected by distinct levels of PA/EE.

## 2. Materials and Methods

### 2.1. Study Population

*The Portuguese Healthy Family Study*, from the Portuguese* Estudo de Famílias Saudáveis Portuguesas* (FAMS), investigates the relationship among metabolic syndrome indicators, physical activity, physical fitness, and body composition in nuclear Caucasian families. Children and adolescents aged 10 to 18 years were recruited in schools from the north and central regions of mainland Portugal and were approached to freely participate in the study with their siblings and parents. Children with chronic diseases (such as asthma and diabetes), physical handicaps, or psychological disorders that might impair their daily routines and physical activities within schools and/or sports clubs were excluded. Given that families with 3 or more children are scarce in the Portuguese population [17], the sample comprises 294 families with only one or two siblings (see Table 1). The ethics committee of the Faculty of Sport, University of Porto, approved the study and written informed consent, and assent was obtained from all subjects.

### 2.2. Data Collection

#### 2.2.1. Physical Activity

Using a 3-day physical activity recall [18], a trained technician interviewed each subject, recording the dominant activity for each 15-min period during 24 h by using a list of categorized activities. Categories from 1 to 9 refer to increasing levels of energy expenditure (METs) of each activity in which category 1 indicates very low energy expenditure such as sleeping or resting in bed and category 9 refers to highly demanding physical work such as high-intensity sports. Approximate median energy cost for each of the nine categories in kcal/kg/15 min was used to compute the daily energy expenditure (DEE) for each individual. The number of 15-min periods for each category was first summed over the 3-day period and weighted by its own median energy cost. DEE was then calculated by summing over the median energy cost of all nine categories and dividing by 3 days. TDEE was then computed by multiplying DEE by subjects' body weights. This method has been previously validated for children and adults [18].

#### 2.2.2. Anthropometric Measurements

The standardized procedures of Lohman et al. [19] were used to measure height with a Siber Hegner anthropometer (GMP instruments), and body composition was measured with a bioelectric impedance scale (TANITA BC-418 MA; Segmental Body Composition Analyser Tanita, Corporation, Tokyo, Japan). Two body composition traits were estimated—%body fat (%FAT) and %trunk fat (%TFAT). This impedance scale has been validated previously with Dual-Energy X-ray Absorptiometry—DXA [20], a gold standard method for body composition measurement. Body mass index (BMI) was calculated as weight (kg)/height (m^2^). Waist circumference was measured at the end of a normal expiration just above the iliac crest, using a nonelastic Holtain tape.

#### 2.2.3. Statistical Analysis

Univariate quantitative genetic procedures as implemented in SOLAR [21] under a special class of the multivariate linear model, namely, the variance components (VC) approach, were used to estimate additive genetic and environmental VCs for each of the BC traits. Prior to all modeling, TDEE, age, age^2^, sex, age-by-sex, and age^2^-by-sex were used as covariates in a preliminary VC model. Residuals were thus derived for each trait and were normalized using an inverse normal transformation, as previously advocated [22, 23]. Heritability estimates (*h* ^2^) were computed using a maximum likelihood approach to estimate variance components under the standard polygenic model as implemented in SOLAR v.4.3.1 software [21].

*Hypothesis Testing*. In order to assess the influence of distinct levels of energy expenditure in body composition genetic regulation we established two main hypotheses.

1. The genetic background of body composition traits is dependent on changes in total daily energy expenditure [TDEE (kcal/day)];
2. The genetic background of body composition traits is dependent on changes in daily energy expenditure [DEE (kg/kcal/day)].

To test for GxTDEE and GxDEE interactions, basic initial hypotheses were formulated regarding the variance/covariance relationship of a BC indicator between family members with different levels of energy expenditure. With regard to GxTDEE and GxDEE interactions, the fundamental null hypothesis is that the expression of a polygenotype (i.e., aggregate of all genotypes related to the expression of a phenotype) is independent of TDEE and/or DEE levels. It can be shown from first principles that if there are no GxTDEE and/or GxDEE interactions, the same BC indicator measured in subjects with different levels of TDEE and/or DEE will have a genetic correlation of 1.0 and the genetic variance will be homogeneous across all levels of TDEE and/or DEE [24, 25]. On the contrary, if GxTDEE and/or GxDEE interactions are present, the genetic correlation will be significantly less than 1.0 and/or the genetic variance will not be the same among all levels of TDEE and/or DEE.

The foregoing requires that we model the variance and correlation as functions of TDEE and/or DEE levels. For the genetic variance function (and similarly for the environmental variance function), we modeled the variance using an exponential function to ensure positivity, which is required since any variance is a squared term [24, 25]: *σ*_*g*_^2^ = exp⁡[*α*_*g*_ + *γ*_*g*_(EE)], where *α*_*g*_ and *γ*_*g*_ are parameters to be estimated. An additional justification for the exponential function is suggested by the alternative name of this approach, namely, the log-linear model of the variance: ln⁡⁡*σ*_*g*_^2^ = *α*_*g*_ + *γ*_*g*_(EE). That is, on taking the natural logarithm of the variance modeled as an exponential function, we have the equation of a straight line. In this form, the variance homogeneity null hypothesis obviously holds for a slope-term equal to 0: *γ*_*g*_ = 0. For the genetic correlation function, we modeled the genetic correlation as an exponential decay function of the pairwise differences in TDEE and/or DEE levels: *ρ*_*g*_ = exp⁡⁡(−*λ*|EE_*i*_ − EE_*j*_|), where *λ* is a parameter to be estimated as a function of the difference in TDEE and/or DEE levels between any two individuals *i* and *j*. Here, we also have an elegant reexpression of the interaction null hypothesis, in this case regarding the genetic correlation, in that a genetic correlation equal to 1 is equivalent to *λ* = 0. This is because for *λ* = 0, we have *ρ*_*g*_ = exp⁡⁡(−*λ*|EE_*i*_ − EE_*j*_|) = *e* ^0^ = 1. At the same time we employed a similar variance function for the residual environment variance as a function of the TDEE and/or DEE environments because it guards against bias in the detection of additive genetic variance heterogeneity. Allowing for variance heterogeneity (i.e., model the variance as a function) in only the additive genetic variance would lead to a bias in the relevant parameter estimate because it is possible in theory for there to be heterogeneity in the residual environmental variance as well. Thus, allowing for heterogeneity in both the additive genetic variance and residual environmental variance can be said to guard against this bias. Also, since our statistical genetic model assumes from the outset that the genetic and residual environmental effects are uncorrelated we did not posit a corresponding environmental correlation function. Allowing for a residual environmental correlation function on the same environmental variable as that for the genetic correlation function would violate the said assumption.

For reasons detailed in Diego et al. [24], the likelihood ratio test statistics (LRTs) to test *γ*_*g*_ = 0 and *λ* = 0 are, respectively, distributed as *χ*_1_^2^ and are as follows: a chi-square random variable with 1 degree of freedom (d.f.) and ((1/2)*χ*_0_^2^ + (1/2)*χ*_1_^2^), a 50 : 50 mixture of chi-square random variable with a point-mass at 0, denoted by *χ*_0_^2^, and a chi-square with 1 d.f. Prior to examination of these hypotheses, however, we first confirmed if the overall GxTDEE and/or GxDEE interaction models provided a better fit to the data than the standard so-called polygenic model. The LRT for these comparisons can be shown to be distributed as ((1/2)*χ*_2_^2^ + (1/2)*χ*_3_^2^) [26]. Under the null hypothesis, the GxTDEE and/or GxDEE models can be thought of as reparameterized models, where the additive genetic variance is equal to exp(alphaG) and the residual environmental variance is equal to exp(alphaE). Taking this into account, on comparison with the polygenic model, the full GxTDEE and/or GxDEE models have three additional parameters, namely, the gamma parameters for the additive genetic and residual environmental variance functions and the lambda parameter for the genetic correlation function. The two gamma parameters give rise to LRTs that are each distributed as *χ*_1_^2^, and the lambda parameter gives rise to an LRT that is distributed as the mixture ((1/2)*χ*_0_^2^ + (1/2)*χ*_1_^2^). The sum of these chi-squares gives ((1/2)*χ*_2_^2^ + (1/2)*χ*_3_^2^).

## 3. Results

The basic descriptive data for TDEE, DEE, and BC traits in fathers, mothers, sons, and daughters are presented in Table 1. Information from 294 families comprising 180 fathers, 253 mothers, 265 sons, and 260 daughters was included. The average family size was 3.3 subjects (Table 2). Total daily energy expenditure follows the expected trend with fathers presenting the highest values, followed by mothers, sons, and daughters, which can be explained by the greater weight of fathers and mothers. These differences are heavily diminished for daily energy expenditure, which does not account for the individuals' weight. However, significant differences were still observed between all classes of relatives for DEE [F(3,795) = 16.126, *P* < 0.001]. As expected, %FAT was higher in females than in males. Sons and daughters' average BMIs were very similar.

Heritability estimates (*h* ^2^) presented in Table 3 were all highly significant (*P* < 0.001), ranging from 0.21 (95% CI: 0.14, 0.37) for %TFAT to 0.34 (95% CI: 0.22, 0.45) for WC meaning that the phenotypic expression of BC traits is in part due to moderate-to-strong additive genetic factors, which is a compelling argument to pursue further specific analysis of their genetic architecture.

The polygenic model was compared to the GxTDEE and/or GxDEE interaction models by means of a log-likelihood ratio test (see Table 4). The GxTDEE and GxDEE interaction models were significantly better than the polygenic model for all the BC traits implying that the GxTDEE and/or GxDEE models fit the data better than the polygenic model for each of these four traits. This means that interindividual variability in the phenotypic expression of these body composition traits is to some degree explained by an interaction between genotype and energy expenditure. As such, different genotype architectures lead to distinct expressions of body composition under the same energy expenditure levels. In Table 5 we present the parameter estimates relevant to interpreting GxE interaction, namely, the gamma and lambda parameters.

Verification of GxTDEE and/or GxDEE interactions was made by comparing both full models to their constrained alternatives for BMI, % FAT, % TFAT, and WC.

**Figure 1:**
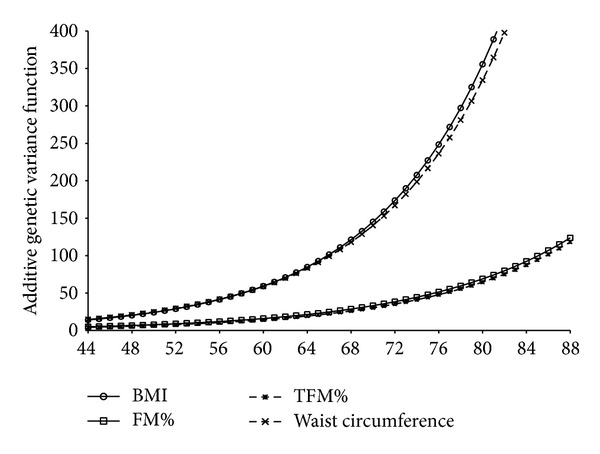
Genotype x Daily Energy Expenditure genetic variance. BMI: body mass index; FM%: fat mass percentage; TFM%: trunk fat mass percentage; WC: waist circumference.

**Figure 2:**
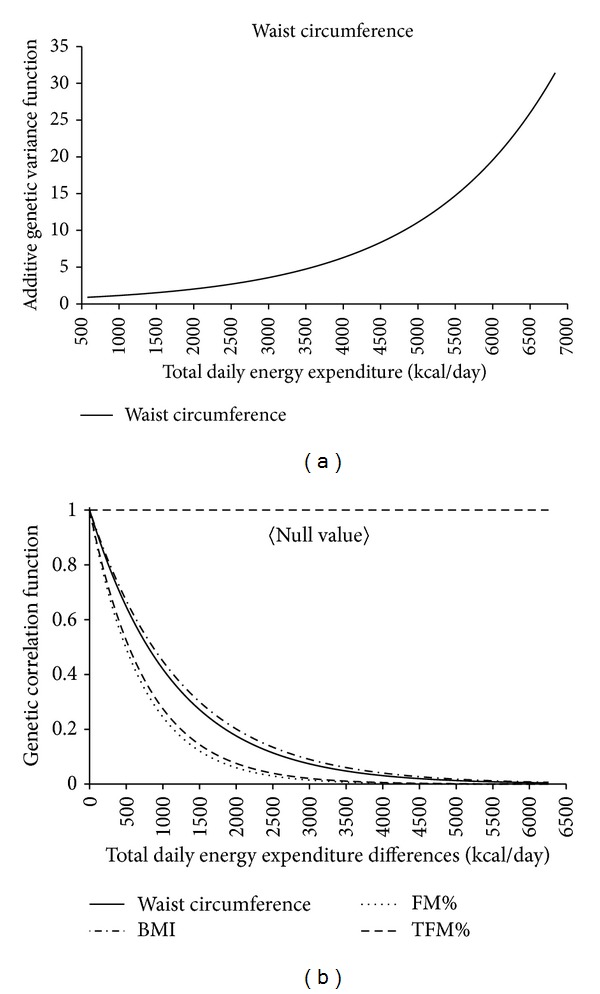
Genotype x Total Daily Energy Expenditure genetic variance (a) and genetic correlation (b). BMI: body mass index; FM%: fat mass percentage; TFM%: trunk fat mass percentage. Genetic correlation function refers to the genetic correlation for the same trait under different TDEE environments.

The significant results for variance heterogeneity and genetic correlation are shown in Figures 1 and 2. All traits were significantly influenced for both GxTDEE and GxDEE models. The significance of the GxDEE model was due to the rejection of the genetic variance (*σ*_*g*_^2^) (Figure 1) homogeneity hypothesis, whereas the significance of the GxTDEE model was due to the rejection of the null hypotheses of a genetic correlation (*ρ*_*g*_) equal to 1 (Figure 2). The only exception was WC in which the genetic variance (*σ*_*g*_^2^) homogeneity hypothesis was also rejected under the GxTDEE model (Figure 2(b)). This means, for instance, despite variance homogeneity for BMI, %FAT, and %TFAT under the GxTDEE model, that a significant interaction with TDEE was still present because the genetic correlation of these traits under distinct TDEE levels was not equal to 1. For example, if the genetic correlation between BMI under TDEE of 2500 kcal/day and a TDEE of 1500 kcal/day is 0.6, then we may speculate that if the TDEE environments differ then different genes are being activated and are being responsible for body composition expression. The null hypothesis of homogeneity in the genetic variance implies a straight line graph (i.e., slope equal to 0) at the level of the natural logarithm of the heritability given that the variances are modeled as exponential functions. Thus, Figure 2(a) shows that the genetic variance does vary as a function of the energy expenditure environment. Specifically, the genetic variance increases with increasing levels of energy expenditure, which means that the higher the TDEE values, the greater the differences in the set of genes activated that are responsible for WC expression. As for Figures 1 and 2(b), the null hypothesis of a genetic correlation equal to 1 is graphically depicted by the horizontal line where the genetic correlation function equals 1. This means that under the null hypothesis the genetic correlation is not to be regarded as a function of differences in the environmental measure. Exponential curves that decay away from the null value simply indicate that the genetic correlation is in these cases a function of differences in the environmental measure.

**Figure 3:**
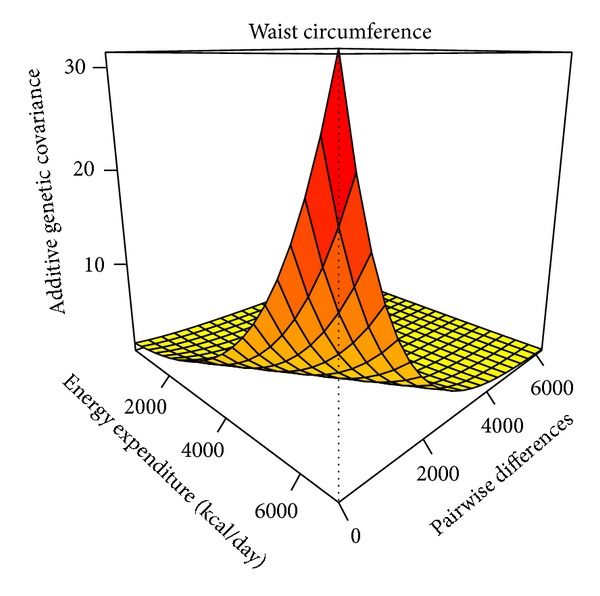
Genetic covariance function for waist circumference under the GxTDEE model.

Figure 3 shows the simultaneous representation of the variance and correlation functions for WC, demonstrating that GxTDEE interaction for WC is a joint function of genetic variance heterogeneity and a genetic correlation different than one. In the figure, pairwise differences refer to the differences between subjects in their TDEE levels.

## 4. Discussion

This study, based on a Portuguese sample of families, aimed to quantify the genetic variance of different BC traits as well as to examine the GxTDEE and GxDEE interactions in modulating the manifestation of these traits in family members. Our results not only confirm the importance of genetic factors in governing the expression of these BC traits, with all* h* ^2^ being significant, but also most importantly showed the importance of both GxTDEE and GxDEE interactions in fat accumulation. To the extent of our knowledge this is the first effort to apply a GxE interaction analysis, using a nuclear family-design study, to test the hypothesis that individual differences in phenotypic expression of BC traits are conditioned by their EE levels; that is, the interindividual variability in different body composition traits is genetically driven and mediated by physical activity exposure.

Body composition heritability estimates reported here were all statistically significant which is in agreement with previous results [27–32]. Waist circumference was the most heritable of the four traits (*h* ^2^ = 0.34), and its value is comparable to the estimates of 0.38 found in the Linosa study [32] and 0.39 found in a study with 533 nuclear families from Spain [28]. The heritability of BMI (*h* ^2^ = 0.25) is lower than those from Spain (*h* ^2^ = 0.44 [28]). The same tendency was observed for body fat with our moderate heritability estimate of 0.25 contrasting with 0.69 in a Swedish sample [33], 0.48 in Nigeria, 0.54 in Jamaica, 0.57 in USA [31], and 0.64 for males and 0.56 for females in a Chinese sample [34]. These discrepancies are usually attributable to different sampling strategies and sample sizes, distinct statistical approaches used to estimate* h* ^2^, and use of distinct adjustments (different covariates). For instance, in our study, all of the* h* ^2^ estimations were controlled for the effect of TDEE which might explain this discrepancy of results. In summary, this wealth of data merely affirms the well-known dictum that heritability estimates are sample specific. Although our* h* ^2^ estimates are somewhat lower than the ones previously reported, we still have from 1/5 to 1/3 of residual variance of BC traits explained by genetic factors, which is a compelling argument to further examine the underlying genetic architecture.

Over the years, researchers have been keen on studying the associations of different environmental exposures with BC [35]. This has mostly been done using a regression-based approach for the detection of phenotypic-level associations between traits among family members [34, 36]. Despite its usefulness in quantifying the degree and sign of the association between distinct BC phenotypes, correlations provide little information regarding the putative mechanisms that underlie such associations. GxE interaction analysis holds the promise of verifying if the association between an environmental factor (e.g., EE) and body fat accumulation is genetically driven, which may be of importance in understanding why people respond differently to physical exercise intervention programs [37].

In the present report, we chose to analyze the potential effects of EE on genotype determination of body composition traits in two different ways: assess the effects of (i) total daily energy expenditure (kcal/min) and (ii) daily energy expenditure (kg/kcal/min). The rationale behind the two different approaches is that TDEE is an absolute measure that is known to be significantly influenced by the effects of age on BMI [38], mainly due to the greater weight of older subjects that is here well observed since there are substantial differences in TDEE between generations, meaning that the differences track with age. Thus, the further analysis of GxDEE allows avoiding the bias related with the influence of greater weight on energy expenditure and a possible age effect on the results of GxTDEE.

The results showed that all BC traits were significantly influenced by both GxDEE and GxTDEE interactions through the rejection of the hypothesis of the genetic correlation being equal to 1 or/and the hypothesis of variance homogeneity. This means that the genotype effects are not exactly the same under different energy expenditure conditions, as they are not fully correlated between distinct DEE and/or TDEE environments. Generally, distinct trends were observed for the two models as the GxDEE interaction was significant due to the rejection of the genetic variance (*σ*_g_^2^) homogeneity hypothesis and the GxTDEE interaction due to the rejection of the null hypotheses of a genetic correlation (*ρ*_g_) equal to 1. Waist circumference was the only trait to be significantly influenced by the two hypotheses and only under the GxTDEE model.

As regards the GxDEE model and the expression of WC under the GxTDEE model, the results presented here show that the genetic variance increases with increasing levels of DEE (and TDEE for WC), which may lead us to speculate that there are genes involved in the expression of body composition traits that are only “triggered” at higher levels of DEE. This particular set of results is not in line with the majority of the previous studies on the interaction between energy expenditure and obesity, in which increasing EE levels have been found to diminish the genetic effects on obesity-related traits. However, the research by Lappalainen et al. [39] also failed to find an association between exercise and the effect of FTO gene on weight changes, in a 4-year followup of 522 overweight or obese subjects randomized to control and lifestyle intervention groups. This evidence poses an argument for the necessity of continuing efforts to unravel the effects of EE at a genetic level that might influence different BC traits. The results under the GxTDEE model indicate that the greater the differences in TDEE levels, the lower the genetic correlations, meaning that the genes influencing body composition traits differ under different TDEE levels. So, in contrast with the GxDEE model, the significance of this model is due to the influence of different genes under distinct levels of TDEE and not to an increase in the additive effects of genes under higher levels of DEE. Previously, physical inactivity was found to upregulate the expression of a number of genes in skeletal muscle tissue in a mice model, which leads to a speculation that the same may be true for obesity markers [40, 41]. In humans, physical inactivity before and after bed rest has been associated with higher levels of tumor necrosis factor *α* (TNF-*α*) [42], which is a potent mediator of gene expression related to inflammation by activating nuclear factor kappaB (NF*κ*B) signalling [43, 44]. On the basis of these data, individuals at different ends of the spectrum of physical activity would be expected to express different sets of genes, one set more associated with subclinical inflammation and the other set less so. In turn, these different sets of genes being expressed across the physical activity spectrum would result in a decay of the genetic correlation away from complete correlation.

GxEE influence on body composition traits has also been studied using DNA analysis [45, 46]. For example, Li et al. [45] genotyped 12 SNPs in obesity-susceptibility loci of 20,430 individuals from the EPIC-Norfolk cohort and reported that each additional BMI-increasing allele significantly increased the risk of obesity in the whole population, but significantly (*p*_interaction_ = 0.015) more in inactive individuals [OR = 1.158 (CI_95%_ = 1.118–1.199)] than in active individuals [OR = 1.095 (CI_95%_ = 1.068–1.123)]. However, in the active group this increase was only 379 g, leading to the conclusion that being active may reduce the genetic predisposition to obesity by 40%. Also, the* FTO* gene was found, when comparing active to nonactive individuals, to have a diminished influence on BMI (0.25 BMI increase per risk allele in active individuals versus 0.44 BMI increase per risk allele in nonactive individuals) and WC (0.64 cm increase per risk allele in active individuals versus 1.04 cm increase per risk allele in nonactive individuals) [46]. More recently, in a robust meta-analysis of 218,166 adults and 19,268 children the results showed that the association between FTO and obesity is diminished by 27% from the effect of PA [13].

This issue is highly challenging and important considering that in many countries researchers and policy makers are trying to deal with the obesity epidemic and associated morbidities not only from a health standpoint but also from a financial view given the public burden in costs of obesity related morbidities [47, 48]. This epidemic has been mostly connected to a fast changing environment (referred to as “obesogenic”) characterized by inducing low levels of energy expenditure and persuasive ways of increasing caloric intake that together constitute a difficult challenge to our genome [45, 46], but our results highlight that genetic adaptability to energy expenditure environments is probably more important than the environment itself. This has been proven previously in a highly cited experimental study with MZ twins [8] in which the variance in response to an overfeeding program of 100 days was three times greater between-pairs than within-pairs for BC traits. The same trend was observed when MZ twins were subjected to an exercise protocol over a 93-day period. Once again, and under controlled nutrient intake, the differences in weight loss were more pronounced between-pairs than within-pairs [10]. Both of these studies substantiate that the more genetically similar individuals are, the more similar they react to the same environment.

We think that our results add to the efforts in trying to disentangle these matters and help to substantiate the latter arguments by suggesting that the phenotypic expression of BC traits is the result of joint effects of genes, EE levels (environment), and their interactions.

Despite the relevance of the present results, some limitations should be acknowledged. Firstly, the sample used may not be representative of the general Portuguese population. Secondly, the method chosen to estimate BC traits, in our case bioelectrical impedance analysis, even though having been previously validated with DXA [20], is not free from bias in its results although the precision of the equipment is ±1%. Nevertheless, this method has been widely used as a BC analyzer in many studies [49–51]. Also, our sample is made of 294 families, which compares with 319 families from the Viva la Familia Study [30] but is somewhat smaller than 533 families from Spain [28]. However, we feel that the joint effects of the size of our sample, the use of state of the art statistical procedures, and the novelty of the analysis in PA genetic epidemiology research are strengths of the present study that warrant consideration.

## 5. Conclusions

In conclusion, the present results showed that the genetic expression of BC traits is significantly influenced by energy expenditure levels. Accordingly, physical activity may be considered an environmental variable that promotes interindividual differences in BC traits through genetic mediation. This is valuable information for health practitioners. More efforts should be devoted to not only identify specific loci that control different BC traits but also test if these loci are regulated or not by different PA levels.

## Figures and Tables

**Table 1:** Sample descriptive characteristics (means ± standard deviations).

	Fathers (*n* = 180)	Mothers (*n* = 253)	Sons (*n* = 265)	Daughters (*n* = 260)
Age (yrs)	45.4 ± 5.2	43.5 ± 4.5	14.7 ± 2.8	14.4 ± 2.8
Height (cm)	170.0 ± 6.7	158.6 ± 5.7	162.2 ± 12.9	156.3 ± 9.7
Weight (kg)	80.1 ± 13.2	66.9 ± 10.2	58.0 ± 16.2	53.6 ± 12.7
TDEE (kcal/day)	3561.8 ± 962.7	2788.4 ± 527.6	2280.6 ± 774.4	2024.9 ± 568.4
DEE (kg/kcal/day)	44.25 ± 8.8	41.92 ± 6.3	39.15 ± 7.4	39.03 ± 10.6
BMI (kg/m^2^)	27.7 ± 4.1	26.6 ± 3.9	21.5 ± 4.1	21.7 ± 3.9
%FAT	23.0 ± 5.7	33.7 ± 5.9	20.0 ± 6.5	27.8 ± 6.2
%TFAT	24.6 ± 6.5	29.9 ± 7.0	16.9 ± 6.8	22.5 ± 7.6
WC (cm)	92.3 ± 10.6	81.0 ± 9.0	72.8 ± 10.4	68.4 ± 8.6

TDEE: total daily energy expenditure; DEE: daily energy expenditure; BMI: body mass index; %FAT: fat percentage; %TFAT: trunk fat percentage; WC: waist circumference.

**Table 2:** Family structures.

	FM4	FM3	FM2	FM1	FM	M3	M2	M1	M	F3	F2	F	2	1	Total
*n*	3	21	105	41	4	7	44	24	2	2	7	1	16	17	294
%	1.02	7.14	35.71	13.95	1.36	2.38	14.97	8.16	0.68	0.68	2.38	0.34	5.44	5.78	100

FM4: father + mother + 4 offspring; FM3: father + mother + 3 offspring; FM2: father + mother + 2 offspring; FM1: father + mother + 1 offspring; FM: father + mother; M3: mother + 3 offspring; M2: mother + 2 offspring; M1: mother + 1 offspring; M: mother; F3: father + 3 offspring; F2: father + 2 offspring; F: father; 2: two siblings; 1: one sibling.

**Table 3:** Heritability estimates (*h* ^2^), standard-errors, and corresponding 95% confidence intervals (95% CI) of the different phenotypes in The Portuguese Healthy Family Study.

Trait	*h* ^2^ (95% CI)	Std. error	*P* value
BMI	0.25 (0.14, 0.37)	0.07	<0.001
%FM	0.25 (0.14, 0.37)	0.07	<0.001
%TFM	0.21 (0.10, 0.32)	0.07	<0.001
WC	0.34 (0.22, 0.45)	0.07	<0.001

BMI: body mass index; %FM: fat percentage; %TFM: trunk fat percentage; WC: waist circumference.

**Table 4:** Results of log-likelihood ratio tests (LRT) and respective *P* values contrasting a polygenic model versus a GxTDEE and GxDEE model for each of the body composition traits.

Trait	Polygenic LnL	LnL	LRT	*P* value
GxTDEE				
BMI	−387.781	−338.660	98.243	<0.0001
%FM	−386.643	−370.572	32.144	<0.0001
%TFM	−387.543	−380.396	14.294	0.002
WC	−380.061	−319.731	120.660	<0.0001

GxDEE				
BMI	−387.781	−356.720	62.124	<0.0001
%FM	−386.630	−378.016	17.227	<0.0001
%TFM	−387.432	−380.609	13.646	0.002
WC	−379.868	−344.650	70.436	<0.0001

BMI: body mass index; %FM: fat mass percentage; %TFM: trunk fat mass percentage; WC: waist circumference; LnL: log-likelihoods; LRT: likelihood ratio test.

**Table 5:** Lambda and Gamma parameter estimates for each of the body composition traits under the GxTDEE and the GxDEE models.

Trait	Lambda*	Gamma*	Lambda LRT	Gamma LRT
GxTDEE				
BMI	0.0008 (0.0004, 0.0017)	—	6.243	—
%FM	0.0014 (0.0008, 0.0026)	—	11.597	—
%TFM	0.0012 (0.0006, 0.0026)	—	7.031	—
WC	0.00009 (0.0005, 0.0014)	0.0006 (0.0004, 0.0007)	11.909	12.711

GxDEE				
BMI	—	0.0896 (0.0733, 0.1076)	—	13.261
%FM	—	0.0731 (0.0525, 0.0949)	—	12.116
%TFM	—	0.0755 (0.0527, 0.0989)	—	9.950
WC	—	0.0868 (0.0710, 0.1043)	—	27.063

*Maximum likelihood parameter estimate followed by the lower and upper bounds for a 95% confidence interval computed following standard methods. BMI: body mass index; %FM: fat mass percentage; %TFM: trunk fat mass percentage; WC: waist circumference; LRT: likelihood ratio test.

## Acronyms

BMI: Body mass index
%TFM: Trunk fat mass percentage
%FM: Fat mass percentage
TDEE: Total daily energy expenditure
DEE: Daily energy expenditure
EE: Energy expenditure
GxE: Genotype by environment interaction
PA: Physical activity
BC: Body composition
WC: Waist circumference.
